# Ethnic inequalities in reproductive, maternal, newborn and child health interventions in Ecuador: A study of the 2004 and 2012 national surveys

**DOI:** 10.1016/j.eclinm.2022.101322

**Published:** 2022-03-06

**Authors:** Paulina Rios-Quituizaca, Giovanna Gatica-Domínguez, Devaki Nambiar, Jair L.Ferreira Santos, Aluisio J D Barros

**Affiliations:** a Facultad de Ciencias Medicas, Universidad Central del Ecuador. Facultad de Medicina de Ribeirao Preto, Universidad de São Paulo. La Armenia, Quito, Ecuador; b International Center for Equity in Health, Universidade Federal de Pelotas, Brazil; c The George Institute for Global Health, Delhi, India; d Facultad de Medicina de Ribeirao Preto- USP, Universidad de São Paulo, Brazil; e International Center for Equity in Health, Universidade Federal de Pelotas, Brazil

**Keywords:** Ethnic groups, Maternal-child health services continuity of patient care, Healthcare disparities, Health care surveys, CI, confidence interval, CVD, national survey of living conditions, ENSANUT, national survey of health and nutrition (encuesta nacional de salud y nutrición), ECLAC, economic commission for Latin America and the Caribbean, ICEH, international center for equity in health, INEC, national institute of statistics and censuses (instituto nacional de estadísticas y censos), LA, Latin America, PR, prevalence ratio, RHS, reproductive health survey, RMNCH, reproductive, maternal, neonatal and children, UBN, unsatisfied basic needs or NBI, (acronym in Spanish) a multidimensional poverty measure, WRA, women in reproductive age

## Abstract

**Background:**

Analysis of health inequalities by ethnicity is critical to achieving the Sustainable Development Goals. In Ecuador, similar to other Latin American countries, indigenous and afro-descendant populations have long been subject to racism, discrimination, and inequitable treatment. Although in recent years, Ecuador has made progress in health indicators, particularly those related to the coverage of Reproductive, Maternal, Neonatal and Child Health (RMNCH) interventions, little is known as to whether inequalities by ethnicity persist.

**Methods:**

Analysis was based on two nationally representative health surveys (2004 and 2012). Ethnicity was self-reported and classified into three categories (Indigenous/Afro-Ecuadorian/Mixed ancestry). Coverage data for six RMNCH health interventions were stratified for each ethnic group by level of education, area of residence and wealth quintiles. Absolute inequality measures were computed and multivariate analysis using Poisson regression was undertaken.

**Findings:**

In 2012, 74.4% of women self-identifying as indigenous did not achieve the secondary level of education and 50.7% were in the poorest quintile (Q1); this profile was relatively unchanged since 2004. From 2004 to 2012, the coverage of RMNCH interventions increased for all ethnic groups, and absolute inequality decreased. However, in 2012, regardless of education level, area of residence and wealth quintiles, ethnic inequalities remained for almost all RMNCH interventions. Indigenous women had 24% lower prevalence of modern contraceptive use (Prevalence ratio [PR] = 0.76; 95% IC: 0.7–0.8); 28% lower prevalence of antenatal care (PR = 0.72; 95% IC: 0.6–0.8); and 35% lower prevalence of skilled birth attendance and institutional delivery (PR = 0.65; 95% IC: 0.6–0.7 and PR = 0.65; 95% IC: 0.6–0.7 respectively), compared with the majority ethnic group in the country.

**Interpretation:**

While the gaps have narrowed, indigenous people in Ecuador continue in a situation of structural racism and are left behind in terms of access to RMNCH interventions. Strategies to reduce ethnic inequalities in the coverage services need to be collaboratively redesigned/co-designed.

**Funding:**

This paper was made possible with funds from the Bill & Melinda Gates Foundation [Grant Number: INV-007,594/OPP1148933].

**Research in context**

**Evidence before this study**

The legacy of colonialism looms large in Latin America, including in Ecuador: the country has among the highest ethnic disparities in development in the region, even as there have been improvements in the realm of health care access in the past decade and a half. Some studies have shown poorer use of health services among ethnic minority populations, although more recent studies have not identified ethnicity as a factor associated with maternal deaths. Overall, prior research on coverage of Reproductive, Maternal, Neonatal and Child Health interventions either does not consider the range of services in the continuum, does not cover Ecuadorian ethnic subpopulations, or does not compare temporal trends.

**Added value of this study**

To our knowledge, this is the first paper to compared the change and inequalities in coverages of six essential RMNCH interventions across ethnic groups (indigenous and Afro-Ecuadorian) in Ecuador in eight years, over a period of time when significant policy shifts were underway (using data from two national surveys, 2004 and 2012). We also assessed socioeconomic factors (wealth, education and place of residence) for each group, and analyzed whether these factors account for the observed inequalities among ethnic groups.

**Implications of all the available evidence**

Our data suggest that ethnic populations still suffer significant disadvantages in terms of coverage of RMNCH services. The implementation of normative guidance and strategies to incorporate intercultural practices, improve participation of indigenous people and improve uptake of services is required. Greater research is needed on the mechanisms by which coverage may have increased and why gaps remain. Moreover, more indicators should be assessed for inequality, for more recent periods of time, and in alignment with indigenous world views.

Alt-text: Unlabelled box

## Introduction

In the global fight against social inequalities in health, as well as the path to achieving the Sustainable Development Goals (SDGs), discrimination on the basis of ethnicity is a prominent barrier – one that should be measured and analyzed systematically and routinely.^1^^,^^2^ In Latin America (LA), the use of an intercultural approach to generate evidence on ethnicity and health to reorient health services is a critical strategy which is part of the first regional policy on ethnicity and health.^3^ An intercultural approach acknowledges with mutual respect that various cultures exist, that this diversity is inherently valuable and that exchange and intergroup dialog in a non-hierarchical framework (that does not privilege one culture or its systems of science over another) is vital for cooperation and co-construction of health and well-being.^4^

This is a break from the legacy of the Latin American region, which has had a long history of colonialization: Latin American populations have for generations been divided into caste-like, racialized categories (e.g., whites, creoles, indigenous, mestizos, mulattos, zambos and afro-descendant). Social inequality across these groups persists in the form of racism and discrimination directly towards indigenous people and afro-descendant populations.^5^ Self-identifying indigenous persons represent 7.2% of the Ecuadorian population^6^ and they have historically experienced exclusion, social marginalization and poverty.^7^^,^^8^ The majority of indigenous people live in rural areas (14.2% vs 2.9% in urban areas); 76% of indigenous households have the lowest supply of drinking water, 12.2% do not have any type of solid waste disposal method, over a third lack conventional telephone service coverage, over three quarters lack mobile phone access, 17.2% face the country's highest rates of overcrowding, and also have the highest rates of female illiteracy among women of reproductive age (17.4%).^9^ In 2018, the proportion of indigenous Ecuadoreans with unmet basic needs was 45.5% compared to 22.2% in the mestizo or mixed ancestry population.^10^

Studies using data from 2004 show that Ecuador is one of the Latin American countries with the greatest ethnic disparities in sexual and reproductive health: in a study by Mesenburg et al. (2018), Ecuador was the country with the lowest coverage in modern contraceptive use, antenatal care and skilled birth attendant out of 15 analyzed countries. In Ecuador, as well as in Bolivia, Perú and Mexico around five out of ten indigenous reported not using any contraceptive method.^11^ Indigenous populations in Ecuador have poorer use of and access to health services, regardless of their economic status,^12^^,^^13^ including reproductive and maternal health care services.^14^ Sahuenza et al., analyzing data from 2014, did not identify ethnicity as a factor associated with maternal deaths, after adjusting for other determinants.^15^ Data for other population level outcomes is un/under-reported.

Ecuador is among a number of Latin American countries that have made significant progress in improving health care access and reducing inequalities in the past decade and a half.^16^^,^^17^ Between 2006 and 2012, as the GDP increased from 4.2 to 12.6%, public investment in the social sector increased,^18^ the Gini index fell about 6.1% (from 52.2 to 46.1), and the proportion of the population living in poverty decreased from 37.6 to 27.3%. Between 2006 and 2014, total health expenditure as a percentage of GDP (Gross Domestic Product) increased from 5.9 to 9.2, and a significant reduction in out-of-pocket spending was observed.^19^ Finally, in the same period, the infant mortality rate decreased from 11.3 (2004) to 8.8 per 1000 live births (2012).^20^ Despite this overall progress, very few studies^21^ have analyzed health reproductive interventions, particularly with attention to ethnicity as a stratifier. We were not able to identify studies that examined whether these inequality reductions have been observed for all ethnic groups, in the spirit of the intercultural approach promulgated by the regional policy in 2017.

Particularly where data availability is high, for instance in relation to Reproductive, Maternal, Neonatal and Child Health (RMNCH) coverage indicators, such analyses are sorely needed. The objective of this study was to fill this gap: we analyzed ethnic inequalities in RMNCH coverage between 2004 and 2012, based on two nationally representative surveys in Ecuador.

## Methods

### Study design and setting

This was a cross-sectional analysis of secondary data; we followed STROBE guidelines (see Supplementary Table 1). Ecuador is an intercultural country located in the northwestern part of South America. Self-identifying indigenous persons were 1018,176, (represent 7,2% of the total population in census 2010), most of them are living in a Mountain and Amazon regions. The Afro-Ecuadorian people number over a million,^6^ and a majority of them live in Coast region . Women and children from these sub-populations were represented in the two nationally representative health surveys we used for the years 2004 and 2012.

### Participant, data sources and study size

The population of interest for the analyses were women aged 15–49 years and children aged under five years in the included surveys. For both surveys, one person per age group was randomly selected: a woman of childbearing age from 15 to 49 years of age for each household, and a child under 5 years of age, according to the methodology established in the official reports.^22^^,^^23^

We analyzed the data of the Reproductive Health Survey (RHS) from 2004, which includes 10,814 women of childbearing age (i.e., 15 to 49 years), and 6140 children under five years,^22^ and compared it with the data of the National Health and Nutrition Survey (ENSANUT, for its acronym in Spanish) from 2012. The ENSANUT 2012 was not the most recent survey but had RHS-corresponding information (more so than, for instance, the National Survey of Living Conditions (CVD, for its acronym in Spanish) from 2014. The ENSANUT 2012 included 18,213 WRA, 5972 children from 0 to 3 years of age, and 10,199 children under the age of five.^23^ The datasets analyzed during this study are publicly available: the RHS 2004 in the repository of the World Bank database and the ENSANUT 2012 in the National Institute of Statistics and Censuses of Ecuador (INEC).^24^ Each survey used multistage cluster sampling to obtain nationally representative data, which is part of official reports^9^^,^^22^ Standardised Demographic and Health Survey (DHS)-style questionnaires were used to collect information from women living in the sampled households. The institutions that carried out the surveys received the relevant ethical approvals; as this is de-identified, publicly available data, ethical approval was not required.

### Measurements of RMNCH indicators

We selected a set of six essential indicators that correspond to each stage of the continuum of care for RMNCH,^25^ using standardized indicator definition criteria^26^ to ensure comparability throughout the four surveys. The selected indicators were: *Use of modern contraceptive, Antenatal care (4+ visits), Skilled birth attendance, Institutional delivery, Early initiation of breastfeeding, and Full immunization.* We calculated coverage prevalence for each intervention and year based of the international standard definitions of the indicators,^26^^,^^27^ their numerators and denominators are presented in the Supplementary Table 2.

### Measurements of dimensions of inequality

We examined inequalities by ethnicity, education, place of residence, and wealth status of the women. We considered three ethnic groups in our analysis: indigenous, afro-descendent, and reference. Since the 2001 population and housing census, Ecuador has identified indigenous populations by spoken language and self-identification. According to international consensus in the LA region,^28^^,^^29^ the "self-identification" criterion has been deemed the most appropriate instrument to assess the magnitude of the indigenous and Afro-descendant population, and there are no significant differences when using the "spoken language". The RHS and ENSANUT surveys analyzed ethnicity through "ethnic self-identification". Therefore, the information is consistent when comparing to the indigenous population from the last census (2010)^30^ and to percentages of women in reproductive age (WRA) who self-identify as indigenous. In the RHS 2004 survey, the indigenous WRA was 7%, whereas the WRA in the ENSANUT 2012 survey was 6.2%.^9^ The previous approach improves the comparability date and reduce the potential bias in the classification of people in the categories in this variable.

Regarding Afro-Ecuadorians, the WRA in the RHS 2004 was 4.7%, and in the ENSANUT survey was 4.3%.^9^ The afro group includes “Afroecuatoriano/afrodescendiente” or “negro/a”. Finally, both surveys includes “indígena” as indigenous group.^22^^,^^23^ The reference group combines groups that in the survey self-identify as: “mestizo” (mixed ancestry), “blanco” (European descent), or others, that include groups like Montubio or other (we were in our analysis unable to consider those who self-identify as "montubios" as a different ethnic group since this was not recognized as a self-identification category in the 2004 survey).

We considered levels of maternal education based on international criteria,^26^^,^^31^ which is done by having low sample sizes at higher education levels and allows comparability between countries. We finally grouped in the national surveys three categories: none, primary education, and secondary education or higher. There is ample evidence that living in rural areas conditions several factors of vulnerability and exclusion from essential health care services.^32^ Place of residence was based on standard definitions and characterized whether the individual was from a household in an urban or rural area.^23^

One simple way of looking at relative poverty is to divide the population into equal quintiles. A key reason for creating wealth quintiles is to look at how equitably RMNCH indicators are distributed by wealth status and each ethnicity group. It is possible to calculate a wealth index and wealth quintiles from any quantitative survey.^33^ The wealth quintiles used in the analyses are based on an asset index, which is pre-calculated in the national surveys. It is estimated through principal components analysis and calculated according to a standard methodology.^34^ The variables used to calculate the score include household assets, access to utilities such as electricity, water and sanitation, and building materials of the dwelling. The households in the surveys are ranked according to the resulting score and split into five equally sized groups (quintiles). Q1 represented the poorest 20% and Q5 the wealthiest 20% of households.

### Statistical analysis

We performed a descriptive analysis of coverage prevalence at the national level by ethnic group (for indigenous people and afro-descendants separately) according to maternal education, area of residence, and wealth quintiles, with their respective 95% confidence intervals. In addition, we created equiplots to show the coverage level and gaps between groups by ethnicity in 2004 and 2012, for each of the six health indicators.^35^

We calculated simple and complex absolute measures of inequality for each indicator and year to examine trends.^36^ Our simple inequality measure was the difference between the indigenous and the reference group (mestizos or whites) alongside 95% confidence intervals. Our complex measure was the "mean difference from best performing subgroup", which takes into account coverage across all population subgroups and their population sizes (i.e. it is a weighted measure)^37^ For both measures, larger values indicate higher levels of inequality, and zero indicates the absence of inequality.

We carried out multivariate analysis using a Poisson regression model. The variables considered for the adjustment in the models were: level of education (secondary or more as the reference group), place of residence (urban as the reference group), wealth quintile (Q5 as the reference group). This statistical approach has proven to be robust and an excellent alternative to logistic regression. As we have a binary outcome, to estimate the Prevalence Ratio (PR) via Poisson regression, we had to calculate the incidence rate ratio (irr) after adjusting the model.^38^ All estimates took into account the survey design and sample weights by using the "svy" estimation command for survey data analysis. For all the analysis, we used STATA 15.0 (StataCorp, College Station, TX, USA) and used the "svy" series of commands, which, along with the robust variance option, guarantees that the assumptions behind the regression model are not violated.^38^

**Role of funding sources:** The funders had no role in the design, analysis, write-up or decision to submit for publication.

## Results

### Socio-demographic characteristics according to ethnicity

In Tables 1 and 2 shown that more than 50% of the women self-identifying as indigenous had attained primary education in 2004, with only a marginal increase by 2012. About 50% of the population of women who self-identified as indigenous were in the poorest quintile, unlike the other two groups, which were relatively more equally distributed across quintiles. This situation did not change considerably in the 8-year period between surveys. The afro-Ecuadorian group had a greater drop in proportion of those in the richest quintile between the years 2004 to 2012.

**Table 1:** Percentage of women according to ethnic group, maternal education and place of residence. Ecuador, RHS 2004 and ENSANUT 2012.

Ethnic group	Year	None			Primary			Secondary +		
		Weighted	%	95% CI	Weighted	%	95% CI	Weighted	%	95% CI
		N			N			N		
Indigenous	2004	128,146	**15.0**	(11.0; 20.0)	483,655	**56.5**	(51.1; 61.7)	244,208	**28.5**	(22.8; 35.0)
	2012	50,311	**15.4**	(11.6; 20.1)	192,978	**59.0**	(53.0; 64.8)	83,674	**25.6**	(21.2; 30.6)
Afro- Ecuadorian	2004	11,306	3.2*	(1.5; 6.6)	166,919	**46.5**	(38.0; 55.2)	180,675	**50.3**	(42.8; 57.8)
	2012	10,344	4.9*	(2.8; 8.3)	98,124	**46.3**	(39.5; 53.3)	103,455	**48.8**	(42.5; 55.2)
Reference group	2004	240,689	**2.4**	(1.9; 2.9)	3511,018	**34.4**	(30.6; 38.5)	6445,111	**63.2**	(59.1;67.1)
	2012	77,006	**2.0**	(1.6; 2.5)	1495,894	**39.4**	(37.1; 41.7)	2228,129	**58.6**	(56.2; 61.0)

Ethnic group	Year	Rural			Urban					
		Weighted	%	95% CI	Weighted	%	95% CI			
		N			N					
Indigenous	2004	699,065	**81.7**	(72.2- 88.4)	156,947	**18.3**	(11.6- 27.8)			
	2012	223,620	**68.4**	(57.0- 77.9)	103,343	**31.6**	(22.1- 43.0)			
Afro- Ecuadorian	2004	123,059	**34.3**	(17.9- 55.5)	235,842	**65.7**	(44.5- 82.1)			
	2012	45,725	**21.6**	(11.3- 37.3)	166,198	**78.4**	(62.7- 88.7)			
Reference group	2004	3644,991	**35.7**	(28.4- 43.9)	6551,823	**64.3**	(56.1- 71.6)			
	2012	1005,590	**26.5**	(20.2- 33.9)	2795,440	**73.5**	(66.1- 79.8)			

**Note:** reference group = mixed ancentry and European descent.

* Sample size < 30 observations.

**Table 2:** Percentage of women according to ethnic group and wealth quintile. Ecuador, RHS 2004 and ENSANUT 2012.

Ethnic group	Year	Poorest			2nd			3rd			4th			Wealthiest		
		Weighted	%	95% CI	Weighted	%	95% CI	Weighted	%	95% CI	Weighted	%	95% CI	Weighted	%	95% CI
		N			N			N			N			N		
Indigenous	2004	443,552	**51.8**	(42.2;61.3)	208,588	**24.4**	(19.7;29.8)	109,222	**12.8**	(8.6;18.6)	66,555	**7.8**	(5.1;11.7)	28,094	**3.3**	(1.7;6.4)
	2012	165,829	**50.7**	(41.8;59.6)	82,244	**25.2**	(20.0;31.2)	44,735	**13.7**	(10.8;17.2)	24,650	**7.5**	(5.3;10.6)	9505	**2.9**	(1.5;5.6)
Afro- Ecuadorian	2004	62,838	**17.5**	(12.3;24.3)	96,564	**27.0**	(21.7;33.0)	79,420	**22.2**	(15.3;31.0)	61,036	**17.0**	(11.1;25.3)	58,414	**16.3**	(11.9;21.9)
	2012	43,546	**20.5**	(15.5;26.8)	48,331	**22.8**	(17.1;29.7)	46,709	**22.0**	(18.8;25.7)	52,493	**24.8**	(17.5;33.8)	20,843	**9.8**	(7.1;13.5)
Reference group	2004	1,350	**13.3**	(10.7;16.3)	1,874	**18.4**	(16.0;21.1)	2,120	**20.8**	(19.8;21.9)	2196	**21.5**	(19.6;23.6)	2651	**26.0**	(22.9;29.4)
	2012	582,853	**15.3**	(13.1;17.9)	723,319	**19.0**	(16.7;21.6)	761,348	**20.0**	(18.5;21.6)	817,359	**21.5**	(19.8;23.3)	916,151	**24.1**	(20.8,27.8)

**Note:** reference = mixed ancestry group; p.p. =percentage points.

### Measures of inequality by ethnic groups

**Figure 1:**
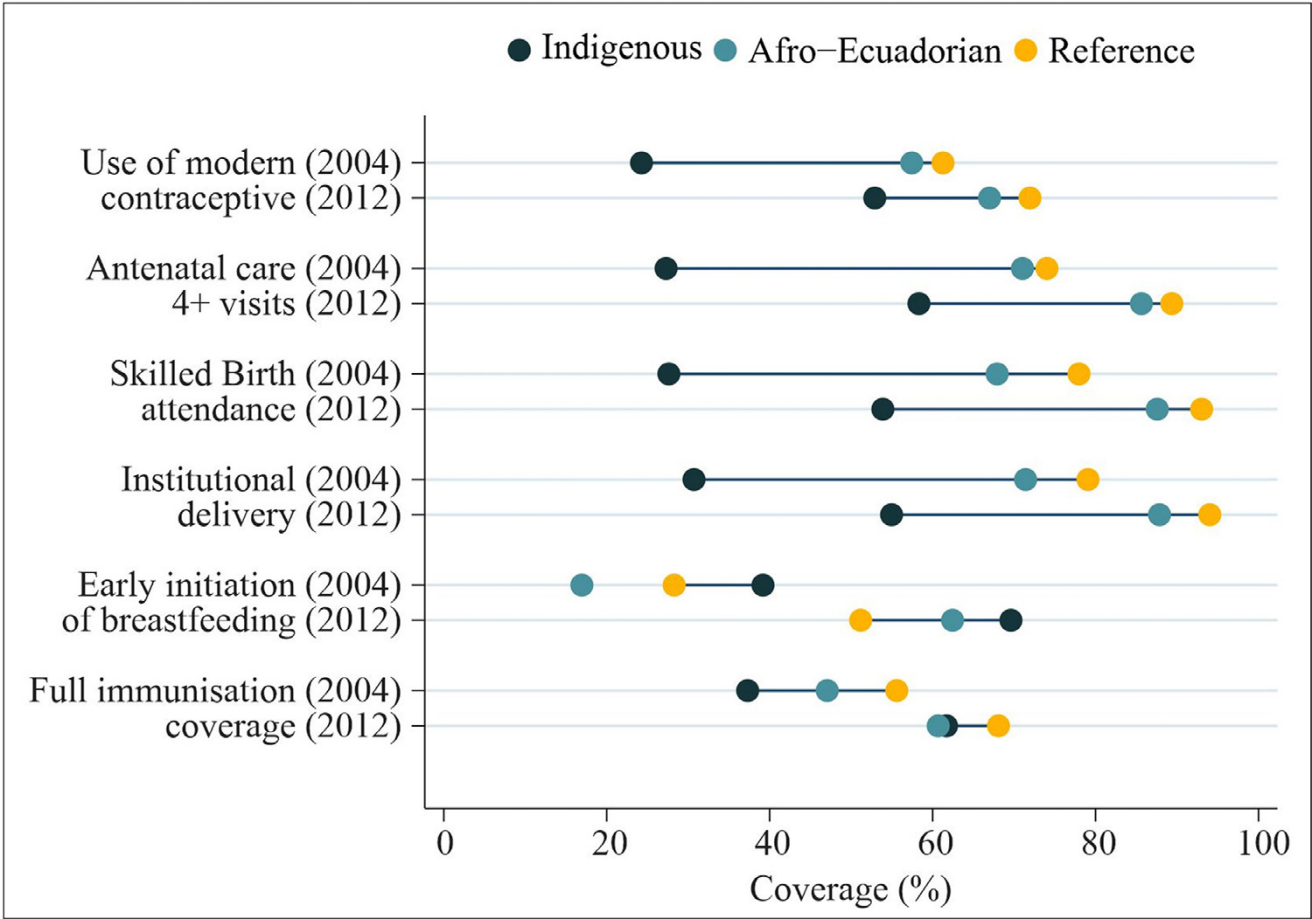
Coverage of RMNCH interventions by ethnic group. Ecuador, 2004 and 2012.

Figure 1 shows the patterns of coverage across RMNCH interventions. In most cases, the magnitude of inequality appears to have declined between 2004 and 2012, although the indigenous group continued to have the lowest coverage. In 2012, full immunization coverage in indigenous and afro-Ecuadorian children was similar, while early initiation of breastfeeding was consistently higher for indigenous groups.

Table 3 shows the coverage and magnitude of inequalities by ethnic group and between years (2004 and 2012) for the six RMNCH interventions. For almost all reproductive and maternal interventions, there are significant differences between indigenous populations and the reference group. The greatest inequality, as measured by difference and mean difference from best was seen in institutional delivery and skilled attendance at birth- which remained even in 2012;

**Table 3:** RMNCH interventions coverage and magnitude of inequalities by ethnic group. Ecuador, RHS 2004 and ENSANUT 2012.

RMNCHintervention	Year	Overall		Indigenous		Afro-Ecuadorian		Reference		DifferenceAfro-Ecuadorian - reference		DifferenceIndigenous - reference		Mean difference from best	
		%	(CI 95%)	%	(CI 95%)	%	(CI 95%)	%	(CI 95%)	p.p.	(CI 95%)	p.p.	(CI 95%)	p.p.	(CI 95%)
Use of modern contraceptive	2004	**58.4**	(56.4;60.4)	**24.3**	(19.5;29.8)	**57.4**	(49.9;64.6)	**61.3**	(59.3;63.2)	**−3.8**	(−12.8;5.1)	**−37.0**	(−44.8;-29.3)	**13.6**	(9.6;17.6)
	2012	**70.9**	(69.2;72.5)	**52.9**	(48.9;56.8)	**67.0**	(58.3;74.7)	**72.0**	(70.2;73.6)	**−5.0**	(−5.3;-4.6)	**−19.1**	(−19.4;-18.7)	**8.0**	(7.8;8.2)
Antenatal care 4+ visits	2004	**69.5**	(66.4;72.4)	**27.3**	(22.4;32.7)	**71.0**	(63.3;77.7)	**74.0**	(50.5;65.8)	**−3.0**	(−11.5;5.5.)	**−46.7**	(−55.5;-38.0)	**16.6**	(12.1;21.2)
	2012	**86.8**	(84.9;88.5)	**58.3**	(50.5;65.8)	**85.6**	(77.8;91.0)	**89.4**	(87.6;90.9)	**−3.7**	(−4.0;-3.4)	**−31.0**	(−31.4;-30.7)	**11.6**	(11.4;11.8)
Skilled birth attendance	2004	**72.0**	(68.6;75.1)	**27.6**	(21.7;34.5)	**67.9**	(56.6;77.4)	**78.0**	(74.9;80.8)	**−10.1**	(−17.3;-2.8)	**−50.3**	(−57.2;-43.5)	**20.1**	(16.6;23.7)
	2012	**89.5**	(88.1;90.7)	**53.9**	(45.6;62.0)	**87.6**	(81.9;91.6)	**93.0**	(91.5;94.3)	**−5.4**	(−5.7;-5.2)	**−39.1**	(−39.5;-38-8)	**14.9**	(14.6;15.1)
Institutional delivery	2004	**73.6**	(70.3;76.7)	**27.8**	(22.0;34.5)	**69.7**	(56.1;80.5)	**74.9**	(70.9;78.5)	**−5.2**	(−10.5;0.1)	**−47.1**	(−52.2;-42.0)	**17.4**	(14.7;20.1)
	2012	**90.2**	(88.0;92.1)	**55.0**	(47.0;62.6)	**87.9**	(82.2;91.9)	**94.0**	(92.4;95.3)	**−6.2**	(−6.4;-5.9)	**−39.1**	(−39.4;-38.7)	**15.1**	(14.9;15.3)
Early initiation of breastfeeding	2004	**29.1**	(25.9;32.5)	**36.4**	(30.2;43.2)	17.9*	(10.0;30.0)	**27.9**	(24.7;31.3)	**−9.9**	(−28.8;8.9)	**8.6**	(4.2;13.0)	**9.0**	(5.5;12.5)
	2012	**52.6**	(49.7;55.5)	**69.6**	(62.7;75.8)	**62.4**	(52.8;71.2)	**51.2**	(48.2;54.2)	**11.3**	(10.6;12.0)	**18.4**	(18.0;18.9)	**8.5**	(8.3;8.8)
Full immunization	2004	**53.0**	(48.0;58.0)	**37.3**	(23.3;53.7)	**47.1**	(26.2;69.0)	**55.6**	(49.9;61.2)	**−8.5**	(−24.7;7.7)	**−18.3**	(−29.5;-7.1)	**8.9**	(2.3;15.6)
	2012	**66.6**	(62.9;70.1)	**61.7**	(50.4;71.9)	**60.7**	(46.6;73.2)	**68.1**	(64.2;71.8)	**−7.4**	(−8.3;-6.5)	**−6.4**	(−7.1;-5.7)	**4.6**	(4.0;5.2)

**Note:** reference = mixed ancestry group; p.p. =percentage points.

We found a reduction in inequality between 2004 and 2012 with the exception of the breastfeeding indicator. The greatest reduction in inequality – as measured by difference – was seen in full immunization, although once a complex summary measure was used, the magnitude of inequality (and of reduction in inequality) reduced. Apart from this, for almost all reproductive and maternal interventions, significant gaps by ethnicity remained in 2012.

Running our Poisson regressions (Table 4), notwithstanding increase in coverage between 2004 and 2012, we found that after adjusting by wealth, education and urban-rural residence area, indigenous women in 2012 had a 24% lower prevalence of modern contraceptive use (PR = 0.76 CI95%:0.70- 0.83), 28% less coverage of antenatal care (PR = 0.72 CI95%:0.64–0.80), and 35% less coverage of skilled birth attendance and institutional delivery (PR = 0.65 CI95%:0.58- 0.73 and PR = 0.65 CI95%:0.57–0.75, respectively), as compared to the reference group. All these differences were statistically significant. The prevalence of early initiation of breastfeeding was 1.15 times higher among indigenous groups compared with children from the reference group. immunization was not found to be statistically significantly higher among indigenous groups as compared to the reference group.

**Table 4:** Crude and adjusted coverage rates of six RMNCH interventions in indigenous and afro-Ecuadorian women and children compared to the reference group. Ecuador, RHS 2004 and ENSANUT 2012.

RMNCH indicator	Indigenous									Afro-Ecuadorian								
	Year	Crude				Adjusted					Crude				Adjusted			
		PR	95% CI			PR	95% CI			Year	PR	95% CI			PR	95% CI		
Use of Modern Contraceptive	2004	**0.40**	0.32	;	0.48	**0.46**	0.38	;	0.56	2004	**0.94**	0.81	;	1.08	**0.96**	0.82	;	1.11
	2012	**0.74**	0.68	;	0.80	**0.76**	0.70	;	0.83	2012	**0.93**	0.83	;	1.05	**0.94**	0.83	;	1.06
Antenatal care 4+ visits	2004	**0.37**	0.31	;	0.44	**0.47**	0.40	;	0.56	2004	**0.96**	0.87	;	1.06	**0.99**	0.90	;	1.11
	2012	**0.65**	0.57	;	0.74	**0.72**	0.64	;	0.80	2012	**0.96**	0.88	;	1.04	**0.98**	0.92	;	1.05
Skilled Birth attendance	2004	**0.35**	0.28	;	0.44	**0.48**	0.39	;	0.59	2004	**0.87**	0.74	;	1.02	**0.88**	0.79	;	1.03
	2012	**0.58**	0.51	;	0.66	**0.65**	0.58	;	0.73	2012	**0.94**	0.89	;	1.00	**0.96**	0.91	;	1.01
Institutional delivery	2004	**0.39**	0.32	;	0.47	**0.51**	0.42	;	0.61	2004	**0.90**	0.76	;	1.07	**0.91**	0.81	;	1.07
	2012	**0.58**	0.51	;	0.67	**0.65**	0.57	;	0.75	2012	**0.93**	0.88	;	0.73	**0.95**	0.91	;	1.00
Early initiation of breastfeeding	2004	**1.39**	1.09	;	1.76	**1.23**	0.93	;	1.63	2004	**0.60**	0.36	;	0.99	**0.65**	0.41	;	1.05
	2012	**1.36**	1.22	;	1.52	**1.15**	1.02	;	1.31	2012	**1.22**	1.04	;	1.44	**1.21**	1.02	;	1.44
Full inmunization	2004	**0.67**	0.43	;	1.06	**0.75**	0.48	;	1.16	2004	**0.85**	0.51	;	1.40	**0.88**	0.55	;	1.47
	2012	**0.91**	0.76	;	1.09	**0.95**	0.79	;	1.13	2012	**0.89**	0.73	;	1.09	**0.90**	0.72	;	1.12

**Note:** PR = prevalence ratio; reference group = mixed ancestry group.

The pattern was similar in the afro-Ecuadorian population relative to the reference group, however, neither raw nor adjusted prevalence ratios were statistically significant in either year, except for the raw and adjusted prevalence of early initiation of breastfeeding in 2012 (PR = 1.22 CI95%: 1.04 −1.44 and PR = 1.21 CI95% 1.02–1.44, respectively).

Early initiation of breastfeeding was reportedly 15% more prevalent in the population that identified itself as indigenous than in the reference population (PR = 1.15 CI95%: 1.02–1.31). This situation was similar in the self-identifying afro-Ecuadorian population, however, the latter did not show statistically significant raw or adjusted values, for both 2004 and 2012 in almost all the indicators analyzed. Detailed results from the adjusted model and other covariates are shown in Supplementary Table 3.

## Discussion

Our study employed secondary data analysis to compare the change and inequalities in coverage of essential RMNCH interventions across ethnic groups in Ecuador over a period of time of eight years (2004 and 2012). We found that comparatively lower educational and wealth attainment among indigenous and afro-descendent populations in comparison to reference groups. While inequalities in service coverage decreased over the period examined, there remained significantly lower coverage among indigenous women, with the exception of exclusive breastfeeding.

As far as we know, this is the first paper that evaluates ethnicity for this broad a range of coverage indicators for Ecuadorian women over a period of time when significant policy shifts were underway. Many findings – such as low educational attainment and high poverty levels among indigenous Ecuadorians - have been seen in other studies.^39^^,^^40^ What is noteworthy is that they have not markedly improved during the eight years studied. The proportion of indigenous women with no education was stagnant at roughly 15% between 2004 and 2012. Conversely, women with greater educational attainment are represented more in the reference and afro-Ecuadorian populations. Nationally, in 2012, while 3.7% of women of childbearing age had no education,^9^ among women who identified themselves as indigenous, this proportion was four-fold higher. Although at the level of the LA region the situation of attendance at educational establishments has improved, the proportion of rural youth who manage to reach higher education and post-secondary education has still not even reached 5%.^30^ The lack of secondary education means that many will have deficiencies in essential life skills like reading and writing and formal job opportunities.^7^ Moreover, in Ecuador 78.1% of the indigenous population works in the informal economic sector and only 11.3% in the formal sector.^41^ This partly explains why 50% of indigenous people remain in the poorest quintile. These indicators are linked and reflect a confluence embedded in a historical burden of social marginality, lack of education and poverty, which cumulatively affect the life course, and unfavorably influence the health of women and children.^42^ Other studies have explored ethic and racial residential segregation demonstrating the link between formal education, socio-economic level and health,^43^ which in turn is an expression of institutionally embedded, structural racism.^44^

Although rural residence was dominant for women who self-identified as indigenous in both time periods, the reduction observed in this percentage between 2004 and 2012, from 81.7 to 68.4%, is suggestive of migration of a greater magnitude in this group, relative to others. Nevertheless, the migration rural to urban areas is not always accompanied by progress: studies show that indigenous rural-urban migrants only have access precarious, poorly paid and low-quality jobs,^45^ associated with family breakdown and loss of community cohesion.^8^ Indeed, in many parts of the world, the situation of the urban poor is worse than that of rural populations overall. There is a need to look at double disaggregation to understand the difference that factors as migration may have on inequalities. It is also essential to stop this migration based on improvements in living conditions - decent education, health, housing, stable job options, among others - in the rural area.

In Ecuador, the period 2006–2014 was characterized by economic growth (with the GDP increasing from 4.2 to 12.6%),^19^ the reduction of poverty, higher public health spending, and the health reform process.^37^ Recent studies carried out in similar periods of time show an increase in health service coverage, including of RMNCH health interventions^16^^,^^17^^,^^36^ and reduction of inequality gaps by wealth and place of residence.^18^ Similar to another study,^21^ the present study also found increasing coverage of health interventions between the years 2004 to 2012 across ethnic groups. Although a reduction in absolute inequality gaps was identified, the pattern of inequality – which continues to disadvantage indigenous population – remained in 2012.

Similar to the finding in Mesenburg et al. (2018), which analyzed 15 countries in LA, the coverage of health service- related RMNCH interventions (Institutional delivery, skilled birth attendance and antenatal care 4+ visits) in Ecuador were the interventions with the greatest differences across ethnic groups, to the detriment of the indigenous population. Further, this study found that 50% of the women who identified themselves as indigenous give birth in their homes. Although it is argued that this phenomenon is justified for cultural reasons since birth and death are family and domestic events^7^; global evidence suggests that the determining factor is the broader context and set of condition in which labor occurs.^46^ Specifically, *skilled attendance at birth* is associated with reduced risk of maternal death and neonatal complications in childbirth and immediate delivery.^47^ Ethnic minorities in LA face a double disadvantage because not only are medical professionals principally available in health centers (where mothers are less comfortable going),^48^ several studies have identified discrimination by health providers against indigenous and Afro-descendant women as a prominent barrier to health service utilization in the region.^49^, ^50^, ^51^ In addition, low ANC attendance among indigenous population has been related to the relatively lower educational level, the higher number of pregnancy and occupation in the agricultural and livestock sector.^52^ Distance, exploitative and restrictive labor relations, and discrimination are key barriers for indigenous women seeking RMNCH services.

Full immunization coverage gaps were reduced considerably for groups of children whose mothers were considered indigenous and afro-Ecuadorian, similar to the study by Messenburg et al.,^14^ although another study, which disaggregates the information at the cantonal level, identifies social inequalities in vaccination coverage for measles.^53^ The decline in inequality observed in this indicator suggests that these efforts to reach this intervention to all ethnic groups have yielded results. This is probably also due to the fact that this intervention was part of the selective package of proposals for developing countries (Selective primary Health care)^54^ which was proposed and applied in the 1990s, and had funding for its execution. What the knock-on implications have been for other service coverage indicators where inequalities have not reduced is a matter of further study.

There are further complexities and nuances worth considering. The context of poverty in indigenous populations residing in rural areas varies further based on geography (for example residence in Amazonian, coastal or mountainous regions). Among the regions of Ecuador, a study by Rios et al. (2021), observed that some provinces of the mountainous region (Chimborazo, Bolivar, Cotopaxi and Imbabura) and several of the Amazon region, have the lowest coverage of interventions in RMNCH. This provinces constitute 40.7% of the indigenous population and while another 24.1% of indigenous Ecuadorians are concentrated in the Amazonian region.^55^ These regions also have other vulnerability characteristics, like high rates of illiteracy, less educated population groups, high total fertility rates, and the lowest rate of doctors per 10,000 inhabitants.^56^, ^57^, ^58^ According to ENSANUT 2012, they also register the highest proportion of births at home (in the rural part) compared to the Ecuadorian coastal. On a positive note, greater prevalence of early initiation of breastfeeding has been identified in provinces of the mountainous region,^16^^,^^17^ and similar to other studies in LA^48^^,^^59^ this coverage was higher for the indigenous children's population, which seems to be more a cultural factor, since indigenous populations do not receive more counseling. Therefore, it is important to explore with an intercultural approach, how factors within each geographic area shape poverty, health seeking and health status – both positively and negatively.^7^

As aforementioned, Ecuador, along with other Latin American countries, approved a policy on ethnicity and health as part of the Sustainable Development Goals 2030 agenda in 2017.^3^ This policy promulgates the need to reorient health services with an intercultural approach to improve the health conditions of indigenous and Afro-descendant peoples. Aligning with, and in some cases even in advance of this important policy, normative documents have been generated for the incorporation of intercultural practices in health services, such as the involvement of traditional midwives in health teams^60^ as well as childbirth initiatives that seek to enhance the meaningful participation of indigenous actors^61^^,^^62^ in order to reduce maternal mortality.^63^ For example, a *Law on Free Maternity and Child Care* (1998–2008),^64^ which devolved fund flows to more readily reach local governments, likely allowed for greater responsiveness of RMNCH services to local needs. This same strategy was further emphasized from 2002 on social participation through users committees comprised of women volunteers,^65^ which might have positively influenced coverage. These initiatives must be more closely understood and evaluated.

In Ecuador, motivated in part by social mobilization and advocacy at various levels, efforts have recently been underway to improve the availability of data for indigenous and afro-descendant people, in order to build differentiated health diagnoses, to monitor health equity gaps that affect these groups.^28^^,^^29^ Nevertheless, national surveys also do not include indicators that may hold greater relevance and meaning to populations themselves, or represent idioms of health that are important to them. There is also a need to expand the complement of indicators,^48^ with those that reflect *Sumak Kawsay* or "general well-being" which is the basis of the indigenous worldview.^66^

This study has some limitations. The inclusion of the "mestizo" group (the result of the intense miscegenation given in Latin American countries) and “montubios” in the reference group,^29^^,^^67^ may have resulted in some under-estimation of coverage, and it does not allow to differentiate the characteristics of these subcategories. In some of the health indicators analyzed, the information on children born in the last 2 to 5 years is used, which will be affected by recall bias. This is, however, non-systematic bias since it is not to a specific ethnic group, so we conjecture that this may not significantly change our results. Some of our population groups of interest are small^68^ and therefore, national surveys are underpowered to look at within-group differences. Despite measurement limitations of our variable on "ethnicity," the two surveys used in this study are comparable since in both years "ethnic self-identification" was analyzed. At this time, this is the most appropriate instrument and comes closest to reflecting population differences, while also allowing comparability over time and between countries.^28^^,^^29^ Another advantage is the comparability of the indicators in both surveys, since they were calculated using a standardized definition of coverage of intervention in all the indicators^26^ thank of the support of at International Center for Equity in Health (ICEH; www.equidade.org).

Further analysis should continue to use the approach of studying inequalities by ethnic group over time, beyond the period assessed in this study. It is likely that inequalities have been accentuated or inflected by the COVID 19 pandemic; which in many cases has affected access to essential health services and has brought to the surface the existing deficiencies of health systems across the region.^69^ This is a critical area for further study and action. Finally, monitoring health inequalities with attention to ethnicity must focus not only on magnitudes of inequality, but also understand the mechanisms, processes and context underlying these inequalities, with involvement of communities facing disadvantage themselves.^70^^,^^71^ This approach will allow research better directed, the development of comprehensive programs and plans to improve health, as well as the improvement of integrated health strategies and medical services according to the general and specific population needs.^72^

In conclusion, similar to the indigenous population of other countries in LA, we found that poverty has remained high in indigenous and afro-Ecuadorian groups. Although Ecuador has had a significant increase in coverage of RMNCH interventions, as well as a reduction in ethnic inequalities between 2004 and 2012, RMNCH coverage remained lower among indigenous people in 2012, regardless of maternal education, wealth and area of residence. It will be important to conduct time series analyzes of health service coverage by ethnic group, as well as in depth research to understand why these inequalities persist. Local research with participatory monitoring could be undertaken that is mindful of local context, social determination,^73^ and indigenous worldviews. Such approaches could better inform strategies, improve the acceptability and accountability of services to ethnic minorities to support their health, well-being and rights. Finally, to achieve improvements in health coverage in countries like Ecuador, where belonging to a certain ethnic group with a burden of social marginality, it is necessary not only to prioritize this group, but also to act on social determinants – improve maternal education, reduce poverty levels and provide local development opportunities through interventions with a broader comprehensive approach.

## Declaration of interests

The authors declare that they have no competing interests.

## Appendix Supplementary materials

Image, application 1
