# Supplementary material for: Ethnic inequalities in reproductive, maternal, newborn and child health interventions in Ecuador: A study of the 2004 and 2012 national surveys
Source: eClinicalMedicine. 2022 Mar 6;45:101322. doi: 10.1016/j.eclinm.2022.101322 (PMC8904232; doi:10.1016/j.eclinm.2022.101322)
Supplement: Supplementary file 1 [file mmc1.docx]

**SUPPLEMENTAL MATERIAL**

**Supplementary table 1.** STROBE Statement—Checklist of items that should be included in reports of ***cross-sectional studies***

|  | Item No | Recommendation | Page No |
| --- | --- | --- | --- |
| **Title and abstract** | 1 | (*a*) Indicate the study’s design with a commonly used term in the title or the abstract | 1 |
|  |  | (*b*) Provide in the abstract an informative and balanced summary of what was done and what was found | 2 |
| Introduction | | | |
| Background/rationale | 2 | Explain the scientific background and rationale for the investigation being reported | 3 |
| Objectives | 3 | State specific objectives, including any prespecified hypotheses | 4 |
| Methods | | | |
| Study design | 4 | Present key elements of study design early in the paper | 5 |
| Setting | 5 | Describe the setting, locations, and relevant dates, including periods of recruitment, exposure, follow-up, and data collection | 5 |
| Participants | 6 | (*a*) Give the eligibility criteria, and the sources and methods of selection of participants | 5 |
| Variables | 7 | Clearly define all outcomes, exposures, predictors, potential confounders, and effect modifiers. Give diagnostic criteria, if applicable | 5-6 |
| Data sources/ measurement | 8* | For each variable of interest, give sources of data and details of methods of assessment (measurement). Describe comparability of assessment methods if there is more than one group | *5* |
| Bias | 9 | Describe any efforts to address potential sources of bias | 6 |
| Study size | 10 | Explain how the study size was arrived at | 5 |
| Quantitative variables | 11 | Explain how quantitative variables were handled in the analyses. If applicable, describe which groupings were chosen and why | 5-6 |
| Statistical methods | 12 | (*a*) Describe all statistical methods, including those used to control for confounding | 6-7 |
|  |  | (*b*) Describe any methods used to examine subgroups and interactions | 7 |
|  |  | (*c*) Explain how missing data were addressed | NA |
|  |  | (*d*) If applicable, describe analytical methods taking account of sampling strategy | 5-6 |
|  |  | (*e*) Describe any sensitivity analyses | NA |
| Results | | | |
| Participants | 13* | (a) Report numbers of individuals at each stage of study—eg numbers potentially eligible, examined for eligibility, confirmed eligible, included in the study, completing follow-up, and analysed | 7-8 |
|  |  | (b) Give reasons for non-participation at each stage | NA |
|  |  | (c) Consider use of a flow diagram | NA |
| Descriptive data | 14* | (a) Give characteristics of study participants (eg demographic, clinical, social) and information on exposures and potential confounders | 8 |
|  |  | (b) Indicate number of participants with missing data for each variable of interest |  |
| Outcome data | 15* | Report numbers of outcome events or summary measures | 8 |
| Main results | 16 | (*a*) Give unadjusted estimates and, if applicable, confounder-adjusted estimates and their precision (eg, 95% confidence interval). Make clear which confounders were adjusted for and why they were included | 8-9 |
|  |  | (*b*) Report category boundaries when continuous variables were categorized | NA |
|  |  | (*c*) If relevant, consider translating estimates of relative risk into absolute risk for a meaningful time period | NA |
| Other analyses | 17 | Report other analyses done—eg analyses of subgroups and interactions, and sensitivity analyses | NA |
| Discussion | | | |
| Key results | 18 | Summarise key results with reference to study objectives | 9 |
| Limitations | 19 | Discuss limitations of the study, taking into account sources of potential bias or imprecision. Discuss both direction and magnitude of any potential bias | 14 |
| Interpretation | 20 | Give a cautious overall interpretation of results considering objectives, limitations, multiplicity of analyses, results from similar studies, and other relevant evidence | 10-14 |
| Generalisability | 21 | Discuss the generalisability (external validity) of the study results | 14 |
| Other information | | | |
| Funding | 22 | Give the source of funding and the role of the funders for the present study and, if applicable, for the original study on which the present article is based | 3, 7, 16 |

*Give information separately for exposed and unexposed groups.

**Note:** An Explanation and Elaboration article discusses each checklist item and gives methodological background and published examples of transparent reporting. The STROBE checklist is best used in conjunction with this article (freely available on the Web sites of PLoS Medicine at http://www.plosmedicine.org/, Annals of Internal Medicine at http://www.annals.org/, and Epidemiology at http://www.epidem.com/). Information on the STROBE Initiative is available at www.strobe-statement.org.

**Supplementary table 2.** Definition of six Reproductive, Maternal, and Neonatal and Child Health (RMNCH) indicators

| **Category** | **RMNCH indicator** | **Indicator denominator** | **Indicator numerator** | **Interpretation** |
| --- | --- | --- | --- | --- |
| Sexual and reproductive health | Current use of any contraceptive method (CPMO) | Women aged 15-49 years currently married or in union | Who are using (or whose partner is using) any method | *Percentage of women between the ages of 15 and 49 who are currently married or in common union, using a modern contraceptive method* |
| Antenatal care | Antenatal care (four or more visits) | Women 15-49, live birth in the last 3/5 years, last-born child | 4+ visit(s) with skilled provider | *Percentage of women aged 15 to 49 years whose last child was born 3-5 years ago, who made 4 or more visits during pregnancy with trained professionals* |
| Delivery assistance | Skilled birth attendance | All live births in the last 2 years | Children delivered by a skilled attendant | *Percentage of live births in the last 2 years that were attended by a skilled attendant* |
| Delivery assistance | Institutional delivery | All live births in the last 2 years | Delivered in a health facility | *Percentage of live births in the last 2 years that were attended in a health institution* |
| Child health | Full immunization coverage | Live children, 12-23/18-29/15-26 months | 3 doses of DPT & 3 doses of Polio & received Measles & received BCG | *Percentage of children up to 26 months of age who received detailed vaccine doses* |
| Breastfeeding | Early initiation of breastfeeding | *Last-born in the last 2 years, women aged 15-49 years* | Put to breast within one hour of birth | *Percentage of last- born who, up to 2 years, put to breast within one hour of birth* |

**Note:** adapted from the indicators’ definition file from the Int’l Center for Equity in Health (www.equidade.org)

**Supplementary table 3.** Adjusted coverage rates of six RMNCH indicators. Ecuador, RHS 2004 and ENSANUT 2012.

**Note:** PR = prevalence ratio; reference group = mixed ancestry.
